# Precedent and the Rule of Law

**DOI:** 10.1093/ojls/gqab007

**Published:** 2021-03-06

**Authors:** Sebastian Lewis

**Keywords:** precedent, null model, rule of law, modes of precedential reasoning, authoritative and persuasive modes

## Abstract

Courts may reason using precedents in various ways, but not all of them satisfy the rule of law. This article provides two ways that are compatible with this ideal and one which is not. In doing so, the article aims to explain the practice of following precedent in law and to offer criteria for evaluating its value. Two claims are defended. First, courts always have a reason to decide precedent-governed disputes by following precedent. This reason is a minimum requirement of the rule of law, and in some cases this reason may be reinforced in the form of an obligation. Secondly, depending on whether courts have a reason or an obligation to follow precedent, two modes of precedential reasoning may be identified. The article explains them in detail. The modes, together with the considerations that are reasons in favour of them or against them, provide a valuable philosophical foundation of precedent-following in law.

## 1. Introduction

Many of our normative practices are influenced by the force that past decisions exert on similar, future situations, in favour of reaching the same result as before. We normally call these past decisions *precedents*, and when it comes to the law we often say that precedents influence the decisions of courts when relevantly similar disputes arise.

But the practice of following precedent is not something we should take for granted. To begin with, one can imagine legal systems where courts do not pay attention, in a normatively significant way, to the ways in which similar disputes have been decided in the past. Similarly, there may be legal systems that prohibit courts from relying on precedents, at least for the purpose of using them as a legal basis for reaching justified decisions. On the other hand, it is not clear whether following precedent has value in its own right, or whether we follow precedent in order to advance other values. Finally, precedents can influence the decisions of later courts in various ways. In the civil law tradition, precedents are often used in order to tip the balance in favour of particular outcomes, but are also used as a means to illustrate how a legal point has been dealt with before. In the common law, by contrast, precedents typically play a more decisive role. In many cases, precedents are authoritative sources of law, in the sense that if the facts in a later case are legally the same as those of a precedent, the later court is often required to deliver the same decision.

My aim is to explain the practice of following precedent in law and provide criteria for evaluating its value. Since there is significant literature on the topic,^1^ I will do this by defending two connected and novel claims. The first argument is that, contrary to what may happen in practice, whenever a precedent-governed dispute arises, the later court *always* has a reason to decide that dispute by following precedent. This reason is grounded on the idea that, by doing so, a legal system has a distinctive way to advance at least the following values: maintaining legal stability, allowing people to rely on reasonable expectations and providing equality in the judicial application of the law.^2^ I will call these values respectively ‘stability’, ‘reliability’ and ‘equality’. Since I take them to be part and parcel of the rule of law ideal, I will refer to them collectively as ‘the rule of law’.^3^ I argue that the principles of judicial transparency and good government strongly militate in favour of courts having to give reasons why they will not follow relevant precedent.

The reason in favour of following precedent is only a minimum requirement of the rule of law. On top of this requirement, a legal system may reinforce its commitment to this ideal by requiring courts to treat the same reason as an *obligation*. Accordingly, the second claim is that we can identify two modes of precedential reasoning precisely by whether courts have a reason or an obligation to decide precedent-governed disputes by following precedent. Consistent with familiar terminology, I will call them ‘persuasive’ and ‘authoritative’.^4^ These modes are not necessarily depictions of what particular courts do, but they provide us with a valuable philosophical foundation for assessing whether what these courts do is desirable or not.

The article is structured as follows. In section 2, I argue that the practice of following precedent should not be taken for granted. To do this, I posit what I will call the ‘null model’, under which courts are authorised to decide precedent-governed disputes without paying attention, in a normatively significant way, to relevant precedent. By showing that the null model is both conceptually possible and we may find arguments supporting it, this offers a useful baseline against which precedent-following can better be assessed. In section 3, I show the extent to which the null model fails to live up to the rule of law. Section 4 argues that one distinctive way to advance the rule of law is by requiring courts to always put in the balance the reason in favour of following precedent. I will argue that, based on a legal system’s commitment to the rule of law, this reason is both non-contingent and content-independent. By showing their main features and differences, section 5 articulates the persuasive and authoritative modes of precedential reasoning. Finally, section 6 offers an assessment of how both modes deal with two evaluative questions: (i) How does each mode advance the rule of law? and (ii) How does each mode avoid replicating substantively incorrect decisions? The purpose of this section is to present some of the tools that are vital for determining whether there is a mode that should be preferred, and for eventually criticising a specific practice of precedent held by courts.

With respect to terminology, I will speak of the ‘earlier court’ to refer to the court that decided a case in the past that is relevantly similar to the one being decided by the ‘later court’. I will also ignore alternative ways of speaking of precedents, such as the *erga omnes* force of judicial decisions,^5^ the constant jurisprudence of courts and the common law. Here, a precedent should be taken to mean a past decision or past case that is relevantly similar to the case at hand. Finally, I will use the expressions ‘relevantly similar’ and ‘legally the same’ as equivalents. The latter stresses the idea that the similarities between the two cases should be relevant in the eyes of the law.^6^

There is one caveat to bear in mind. My account, like that of many others,^7^ does not depend on a strict separation between vertical and horizontal *stare decisis*.^8^ As I will explain, I think this distinction is useful to capture those specific precedents that, for various considerations, certain courts *must* follow. But that question is secondary to determining whether later courts *always* have a reason to follow precedent—the question that motivates this article.

## 2. The Possibility of the Null Model

Justice and courts have always had a complex relationship. John Gardner wrote that ‘judges should first and foremost administer justice’,^9^ and before him HLA Hart held that we naturally think of justice as being administered according to law.^10^ This complex relation has contributed to the way we have historically called courts, shifting between courts of *justice* and courts of *law*.^11^ Today it is not controversial to say that, when courts decide disputes, and thereby attempt to deliver justice, they do so by applying the law to the case at hand.

But what is the specific law that would allow judges to reach legally justified decisions? The answer to this question inevitably varies across different legal systems, as it depends on the sources of law in the particular legal system under consideration. Legislation is typically a source of law, meaning judges can reach legally justified decisions by applying statutory law. But things are more complex when it comes to ‘case law’. In the common law, judicial decisions are generally given legal status,^12^ meaning courts can reach legally justified decisions by applying case law to the dispute at hand.

The same cannot be said, or at least not as simply,^13^ with respect to various countries in the civil law tradition. In many of these countries, it is generally the case that judicial decisions are authoritative with respect to the parties to the dispute *only*, and this effect is commonly known as the ‘relative force’ of judicial decisions.^14^ This means, first, that as a general rule,^15^ only the parties to the dispute may benefit from the authoritativeness of the decision—for example, to demand conformity with its terms. (To this extent, the relative force of judicial decisions is also present in the common law.) But, secondly, if a relevantly similar dispute arises between *other* parties in the future, the later court—in the civil law—may be prevented from reaching a legally justified decision *solely* by following precedent. As Eva Steiner writes concerning French law: ‘Explicit reference by a court to its own *jurisprudence* when giving a decision and, more generally, citation of previous cases is not allowed when these are meant to serve as a legal basis for the court’s decision.’^16^

Of course, French law does not exhaust the civil law tradition. The point is that, often, the relative force of judicial decisions entails that precedents are not generally given legal status. As Merryman and Pérez-Perdomo observe: ‘[in the civil law] prior judicial decisions are not “law”’.^17^ Therefore, in order to reach decisions justified by law, civil law courts usually rely on undisputed *legal* sources. Legislation, again, presents itself as a good example of an undisputed source of law, but in some jurisdictions the constitution also serves as a legal basis—as well as ratified international treaties. For this reason, if a court relies on a legal source, but in doing so it ignores or contravenes relevant precedent, the decision may nonetheless be legally justified.^18^

But if precedents may not provide justificatory basis whatsoever for reaching legally justified decisions, what normative role do they play? Drawing from a comparative analysis including both civil law and common law jurisdictions, Aleksander Peczenik concluded that precedents typically perform the following roles: (i) they bind formally; (ii) they have normative force but do not bind formally; (iii) they are neither formally binding nor have any normative force, but provide ‘further support’ (see note 19); and (iv) they serve to illustrate points of law.^19^ Peczenik’s conclusion makes room for the proposition that the existence of a relevant precedent may make no normative difference in the decision of a later court. This conclusion is supported by the fact that, as we saw before, many civil law courts may reach legally justified decisions *even if* they have contravened or otherwise ignored relevant precedent.

It is true that the civil law is changing towards giving precedents a more decisive role (see note 13). That has been because of the long and arduous work of courts, the legal community and the legislatures. But this change reveals a point of departure: the possibility that the existence of a precedent may make no normative difference in the decision of a later court. This possibility provides the conceptual basis for thinking that a particular model of precedent may exist. I will call it the ‘null model’, because for a later court the existence of a precedent regulating the issue at hand makes no normative difference to the vital question of how such an issue should be decided. This type of case should be distinguished from those in which the later court offers reasons for not following relevant precedent. In this situation, the precedent makes a normative difference, since the later court has to meet a justificatory burden for failing to conform to it. The null model, by contrast, seeks to capture those situations in which the existence of a relevant precedent is another non-normative fact of the world: the precedent does not even tip the balance of reasons in favour of reaching the same outcome as before. These could be, for instance, cases where the later court knows about the existence of a precedent, but it does not show why the precedent will not be followed.

Various reasons may support having the null model. With some adjustments, an advocate of this model could rely on the following. First, legal systems have, all else being equal, an interest in preventing judges from making creative innovations in the law. In order to keep the law pure, the influence of judicial decisions on future cases should be limited as much as possible, and the null model would be one way of doing this. Secondly, judges have a duty to treat parties impartially and objectively. Since the practice of following precedent is normally affected by factors external to the law, such as the prestige of the earlier court, these factors may introduce bias in the court’s decision. In order to avoid this, judges should not follow precedent. Finally, the law should have a democratic character. Precedents are often given legal status, but they are not created by a democratic process. One way to avoid this non-democratic upshot is by giving judicial decisions relative force only, thus restricting the influence of precedents on later courts.

The possibility of the null model is valuable for two reasons. It reminds us, first, that the values brought by judicial conformity to precedent, soon to be studied, should not be taken for granted. The null model, as such, is a way to appreciate these values by conceiving of a legal system where the same practice does not exist. Secondly, the null model confronts us with the following challenge. If nothing in law’s nature prevents courts from lawfully adjudicating disputes under the null model,^20^ then why doesn’t any legal system have it—at least officially? Put differently, what causes many later courts to pay attention, in a normatively significant way, to the ways in which earlier courts have decided relevantly similar cases?

## 3. The Rule of Law

Many ideas are usually associated with the expression ‘the rule of law’. It is often said, for example, that the rule of law is first and foremost a requirement of good governance. People exercising positions of political authority should be subject to mandatory rules aiming to minimise the potential for arbitrary government and even tyranny.^21^

The rule of law is also identified with a set of formal demands that seek to allow individuals to better anticipate what the law may require from them. Accordingly, the standards by which the law will guide conduct should be clear, public, general, prospective and stable, so that people may know in advance how to plan their lives under the law.^22^

Similarly, the rule of law requires minimum procedural conditions that courts should observe when they administer justice, in order to ensure parties a ‘fair hearing’.^23^

Finally, the rule of law is also associated with substantive conditions for the existence of the law. Some say, for example, that the law should meet basic demands of fundamental rights.^24^ Others claim that the rule of law should go one step further and secure basic social rights.^25^

These various ways of conceiving of the rule of law have led many scholars to distinguish between ‘formal’ and ‘substantive’ versions of the rule of law.^26^ As Brian Tamanaha notes, ‘formal theories focus on the proper sources and form of legality, while substantive theories also include requirements about the content of the law’.^27^ However, as Tamanaha also writes, though the distinction is informative, it should not be taken at face value. The reason is that ‘formal versions have substantive implications and the substantive versions incorporate formal requirements’.^28^ Other scholars have shared similar worries.^29^

Importantly, for the purposes of this paper, I will adopt a modest understanding of the rule of law—one usually associated with formal versions. The reason is not because I reject substantive versions of the rule of law—a point we need not settle here. The point, rather, is that this modest version is sufficient to ground precedential constraint, while avoiding the profound disagreement entailed by many of the substantive versions. In effect, the more substantive one’s conception of the rule of law becomes, the more disagreement it seems to produce.^30^ On the other hand, ‘All substantive versions of the rule of law incorporate elements of the formal rule of law’.^31^ In sum, certain formal demands are necessary conditions of the rule of law, while also being sufficient to ground precedential constraint. This is the moderate strategy I shall pursue.^32^

In particular, there are three rule-of-law values the advancement of which gives us reasons to reject the null model, and prefer instead one of the modes that I will propose.^33^ These are the values of stability, reliability and equality in the application of the law.^34^ I will briefly explain these values, and will then show the extent to which the null model runs counter to them.

### A. Stability and Reliability

Stability is a state of affairs in which the content of the law of one country is settled over a considerable amount of time. The proviso of time is important because it would not seem plausible to think that a legal system has achieved stability in relation to a specific matter if the content of the law on that matter changes too frequently. Achieving stability is a matter of degree, for there is no threshold indicating how much time is required for the content of the law to become stable. This depends on many factors, such as the area under examination, where in some cases one specific change in the content of the law may have a much more systemic impact than changes in other, less sensitive areas.

It is not clear whether stability has value in its own right.^35^ It seems that we value stability not for the sake of stability, but because it is instrumental to the realisation of other values, in particular one which directly bears on the decisions we make in our daily lives: the value of reliability. It is valuable that people know what the content of the law is, what the law commands, allows and forbids, for when people have this knowledge, they can rely on it as valuable information to adopt decisions that will impact their lives. In many ways, people can shape their lives, anticipate events and be psychologically confident thanks to the structural boundaries offered by the law. But in order for the law to allow this, the law cannot suffer changes too frequently. The law, in other words, needs to be stable. Stability and reliability are thus two sides of the same coin: what may advance the former may also promote the latter, and what may undermine the former may also affect the latter.

Since stability and reliability are intimately connected, I will focus on reliability to argue against the null model. Recall that under the null model the fact that a precedent exists—regulating the matter at hand—does not even tip the balance of reasons in favour of reaching the same decision. True, if the case is governed by statute and the application of the relevant norm is straightforward enough, then people have a reason to believe that such a case should be decided in the way prescribed by the statute (assuming, of course, that courts have a duty to give effect to the content of statutes). But where the application of the relevant norm entails a ‘hard case’, this reason for belief might become weaker, or even disappear, if the court adjudicates under the null model.

Take cases of statutory vagueness, or where there are various candidates for a correct interpretation of the statute, or where applying the norm would conflict with a moral consideration. For our purposes, a hard case is one where there are good reasons to believe that the court may arrive at different conclusions concerning the statute’s application. All these outcomes, further, are justified by law. In a hard case, the null model entails a probabilistic lottery. If there are two correct ways of deciding the case, but the fact that one of them is supported by precedent does not count in the balance, then *ceteris paribus* parties have a 50% chance of anticipating the court’s decision rightly. This number decreases when the amount of possible justified scenarios increases. If, say, there are four possible correct answers, the chances of parties getting things right is 25%, again *ceteris paribus*.

Provided that there are alternative modes of adjudicating precedent-governed disputes that can offer more reliability to parties, this probabilistic lottery runs counter to the rule of law. As I will argue, one of these ways is by making it the case that courts *always* have a reason to decide precedent-governed disputes by following precedent. The existence of this reason increases the degree of reliability given to parties *vis-à-vis* the null model. Parties can rely before litigation on the fact that courts always have a reason to decide disputes in conformity with relevant precedent. But since this reason does not exist in the null model, parties are left to whatever assessment of the correct scenarios the court deciding the case makes.

### B. Equality

Equality is a contested notion, and some have shown scepticism about whether there is something distinctive in equality other than the generality presupposed in every norm-application.^36^ But the existence of a norm is one thing; its application is another. Thus, for reasons external to the norm itself, there can be cases that fall within the norm’s scope, but which may receive a different treatment—despite the fact that in virtue of the norm’s generality they should not.^37^

This difference of treatment can be explained by the discretionary element entailed in the application of a norm. To begin with, applying a norm requires determining, and eventually justifying, whether certain facts of the world fall within the norm’s scope. This process is generally known as *subsumption*, and though the margin for discretion might be reduced, adjudication will often require sensible judgment. It is here where judges come into play. Judges are trusted with the power to determine, *inter alia*, whether facts before them can count as instances of the norm’s material scope of application. If they count, then we often say that judges have an obligation, though not necessarily a conclusive one, to decide the case according to the result provided by the norm.^38^

Subsumption can be a straightforward or complex process, depending on whether it is clear or disputed that the facts of the case fall within the norm’s scope. When it is clear, we can think of the case, at least in respect to subsumption, as an easy one. But subsumption does not exhaust the adjudicatory work of judges: they still have to determine whether the norm should be applied all things considered. As Kenneth Winston observes, judges may ‘refrain from applying a law to a case that it clearly covers, for example, on the ground that there are features to the case that were not anticipated’.^39^ Conversely, complexity in subsuming facts is a reason for the case to be seen as a hard one.

As it relates to precedent, equality requires courts to limit the menu of possible correct answers that, absent relevantly similar cases decided before, they would otherwise have. This means that, if the earlier court reached a decision that was initially justified, the later court is prevented from reaching a different decision without justification. For example, suppose it was an open question for an earlier court whether to treat a particular object as an instance of the statutory term ‘wheelchair’. In a relevantly similar case, a later court would thus not be able to say, at least not without justification, that the same object is not a wheelchair had the earlier court decided that it was.

But equality, in this sense, can also play a role in the equitable dispensation of legal requirements—assuming, of course, that courts have the corresponding power. If, on grounds of equity, an earlier court had made a particular exception in favour of a party, then the later court is prevented from denying the same exception without justification.^40^

The null model authorises courts precisely to dispense with this justificatory requirement—namely, of having to argue why they will depart from the decision of the earlier court. The final decision, which affects equality but does not offer a justification, is legally justified. Contrary to this state of affairs, as I have argued, equality requires later courts to give reasons why they will depart from past decisions. I have considered the examples of legal interpretation and equitable dispensation, but these do not exhaust the adjudicatory work of judges.

In sum, the null model can be lawful, and supported by various reasons, but there are important reasons as well for thinking that it fails to live up to the rule of law. And the rule of law is an ideal worth pursuing. It has been said, for example, that the rule of law is ‘law’s own virtue, respect for which is needed for the law to have any other virtue’.^41^ Others think of it as ‘the specific virtue of legal systems’.^42^ Still others see it as ‘the morality that makes law possible’.^43^ These various ways to think about the rule of law suggest that a legal system has important reasons to live up to this ideal, including stability, reliability and equality. These reasons might not outweigh all competing values, as some scholars have rightly noted,^44^ but, all else being equal, they do recommend a commitment to the rule of law. In virtue of this commitment, departures from the rule of law, as Tom Bingham argued, should have a ‘clear justification’.^45^

In the next section, I will lay out the structure for articulating two modes of precedential reasoning that, unlike the null model, advance the rule of law.

## 4. A Fresh Start

Having argued against the null model, it is time to propose a fresh start. Unlike before, where I proceeded bottom-up,^46^ this time I will proceed top-down. I will start from the rule of law and then articulate two modes of precedential reasoning that are consistent with this ideal.

The argument in this section is as follows. The first subsection argues that one distinctive way to advance the rule of law is by requiring courts to always decide precedent-governed disputes by following precedent. Notice that the claim here is a modest one. I will not be arguing that courts should always have an *obligation* to follow precedent, but only a *pro tanto* and non-contingent reason. Precisely because of this caveat, the claim is, I think, novel. What is needed, in order to establish the non-contingency of the reason in favour of following precedent, is the normative backup of a positive second-order reason.^47^ In the second subsection, I will argue that a legal system’s commitment to the rule of law can do this work. The upshot is that the non-contingent reason to follow precedent is a minimum requirement of the rule of law.

### A. Advancing the Rule of Law

The task before us is to show that the reason to follow precedent is a valuable means to advance the rule of law. I do not think, based on the relevant literature,^48^ that this claim is controversial. But it is important not to take it for granted—observing also, as others have done,^49^ that sometimes following precedent *may not* necessarily advance the rule of law. This is why a *ceteris paribus* clause is needed: all else being equal, following precedent is a distinctive means towards a valuable end.

Consider stability and reliability. Both values demand that the content of the law remain stable over time, so that people can rely on it to adopt decisions. Since stability is instrumental to reliability, think of the problem in the following way. If the precedent and present cases are legally the same, but they receive different treatment, can people form reliable expectations concerning the ways in which these types of cases will be decided? Possibly yes, if such a decision is treated as an exception.^50^ But where there is a systematic and widespread practice of giving a different treatment to two cases that are legally the same—the past and present cases—people might have a reason *not to* form any expectations whatsoever. When courts follow precedent, people are given a reason to believe in, and thereby rely on, the fact that if their cases are relevantly similar then they will receive the same treatment.

Sometimes, however, courts will have to depart from past decisions, and this is particularly true with respect to those decisions that are notoriously suboptimal—ie are notoriously unjust, arbitrary or otherwise unsound. Admittedly, not every suboptimal precedent ought to warrant a departure; otherwise, the contribution of the practice of following precedent to stability and reliability may be jeopardised.^51^ This raises the question of whether there is a threshold for not following precedent.

It seems that, if a legal system wants to balance tailored justice (to the parties) with stability and reliability, that threshold will unavoidably be affected by what Andrei Marmor called ‘moral vagueness’.^52^ The threshold, in other words, will contain an evaluative concept whose application requires a value-laden judgment by the later court.

Take the House of Lords’ 1966 Practice Statement: precedents of that court are ‘normally binding’ (on the same court), but they admit departures ‘when it appears *right* to do so’.^53^ It seems difficult, and perhaps self-defeating, to anticipate all the necessary and sufficient conditions for establishing when it is right for that court to depart from precedent.^54^ For this reason, a legal system will most likely delegate this determination on judges themselves, who will balance tailored justice with stability and reliability, often leaning towards the latter—as the same House of Lords (today the UK Supreme Court) has shown over time.^55^

Concerning equality, we could ask a similar question. Could an observer affirm that courts in a particular country are being consistent with past exercises of judicial discretion when they adjudicate differently in disputes that are legally the same? Again, possibly yes, if these decisions are exceptional; but not if they count as the general rule. The practice of following precedent aims to prevent the passage of time from making a difference to the treatment of two disputes that are relevantly similar. Put differently, were a judge required to adjudicate simultaneously two disputes that are legally the same, she should decide them in a like manner. But judges seldom adjudicate disputes simultaneously; rather, they do it over a period of time. The practice, therefore, aims to prevent the passage of time from altering this kind of treatment. Accordingly, when two disputes are legally the same, but they arise at different times, one way to deliver consistent treatment is to decide the new case in conformity with the precedent.

Conformity to precedent is a *distinctive* means to advance the rule of law. By precedent-following, a legal system can foster this ideal in ways that can only be done through adjudication—for example, by tackling specific problems raised by litigation among individuals, resolution of which has systemic implications for the legal community as a whole. The null model, by contrast, is precisely characterised by the fact that the legal system has decided not to live up to this distinctive opportunity—of advancing the rule of law via precedent-following.

### B. Commitment to the Rule of Law

I have argued that the values of stability, reliability and equality are ends towards which the practice of following precedent presents itself as a distinctive means. If my discussion is sound, it provides a *pro tanto* reason for judicial conformity to precedent. That is, to the extent that courts have a reason to advance the rule of law, they also have a reason to follow precedent.

But more needs to be done. In particular, it is vital that the reason to follow precedent does not depend on whether a later court agrees with the precedent’s correctness. Otherwise, we face two crucial objections: one descriptive, the other normative. The descriptive objection would argue that many courts follow precedents independently of their correctness.^56^ The normative objection, by contrast, would insist that, if precedent-following is to make a contribution to the rule of law, it should not depend on a later court’s sympathy with the precedent.^57^

I shall pursue the opposite strategy: the reason to follow precedent is content-independent, in the sense that later courts have it whether they agree with how the precedent was decided or not. One challenge that arises immediately is the following: how can we justify later courts that *knowingly* replicate incorrectly decided precedents? How can an agent be justified in performing an action she knows to be incorrect? Drawing from contributions on argumentation theory^58^ and practical reasoning,^59^ I will argue that a legal system’s commitment to the rule of law can justify later courts in following precedents they know to be wrong.

To illustrate the problem, it will be useful to distinguish between two versions of following precedent: ‘weak’ and ‘robust’.^60^ According to the weak version, a later court has a reason to follow precedent *only if* the precedent was correctly decided. By contrast, in the robust version a later court has such a reason *independently* of the precedent’s correctness. On this view, the mere fact of a precedent’s existence—regulating the case at hand—is a reason for the later court to follow it. It is the robust version that Frederick Schauer has in mind when he argues: ‘if we are truly arguing from precedent, then the fact that something was decided before gives present value despite our current belief that the previous decision was erroneous’.^61^

For Schauer, precedent-following entails the robust version. Yet the problem with this approach is that an incorrect precedent cannot become correct simply by virtue of being followed in the future. If I lied to you in the past, lying to you again will not be justified simply because I already did it once. The reason, in general, is that the mere performance of an action is not sufficient to alter its moral quality.^62^

A similar situation happens in law. If an earlier court set a precedent that the later court knows to be wrong, following it will not make the later court’s decision right. Or not unless further premises are added to the normative background.^63^ A typical premise of this kind is a positive second-order reason for action.^64^ In general, first-order reasons are considerations in favour of performing or refraining from performing a certain action. Positive second-order reasons are considerations in favour of *acting on* one of these first-order reasons. Negative or ‘exclusionary’ second-order reasons are considerations supporting *not acting on* certain first-order reasons.^65^ The combination of a first-order reason plus an exclusionary second-order reason is what Joseph Raz calls a ‘protected reason’.^66^ (I will return to this idea in the next section.)

Commitments are a good example of a positive second-order reason. If, for instance, one has good reasons to pursue a particular project, a commitment can be a second-order reason to act on these reasons. More importantly, sometimes this commitment can be the sole reason for why one perseveres on the chosen path—say, because one’s initial reasons no longer exist, such as when one’s preferences, or the circumstances of life, have changed. In these cases, commitments provide, as Ruth Chang writes, ‘the grounds for *new* will-based reasons’.^67^ Similarly, in virtue of a particular commitment, sometimes we see ourselves making decisions we would not otherwise make. Your commitment, say, to a professional tennis career can put you in a situation where every year you miss the birthday party of your daughter, because every year you must play the same tournament in another country. Without this commitment, it would be wrong to miss your daughter’s birthday. In more dramatic scenarios, as Raz writes, ‘one’s choice does make it right for one to pursue a goal which but for one’s commitment to it would have been a wrong goal to pursue’.^68^

As a positive second-order reason, therefore, a commitment is a premise that can be added to the normative background to justify what would otherwise be unjustified. Likewise, when it comes to precedent-following, commitments can do a similar work. Suppose two years ago your daughter Claudia turned 15 and you allowed her to drink wine for the first time. In about a week, Peter, your youngest son, will reach the same age, and he wants to know whether you will allow him to drink wine as well. Assume, further, that with time you have come to realise that it was never right to allow Claudia to drink wine at such a young age. That decision was, in other words, a mistake. Now, if the situation of Peter is relevantly similar to that of Claudia,^69^ do you have a reason to allow Peter to drink wine?

Again, according to weak precedent-following, you do not have such a reason if it was wrong to allow Claudia to drink wine. Now, suppose you have a commitment not to disappoint the reasonable expectations that your past decisions have created in your children. Or assume that it is really important for you not to give your children the impression that you treat them differently when they are similarly situated. Thus, unless *very* exceptional circumstances arise, you have a commitment to show them that you treat them equally by following your past decisions.

This type of commitment can ground robust precedent-following. It can give you *now* a reason for following a past decision you know is incorrect. Similarly, a legal system’s commitment to the rule of law can justify those courts that follow precedents known to be wrong. As Neil Duxbury writes, ‘Even a decision widely considered wrong might continue to be followed if people have reasonably relied on it arranging their affairs’.^70^

Therefore, if a legal system has a commitment to the rule of law, and if judges live up to that commitment,^71^ then robust precedent-following is a distinctive way in which they could do so. Now, we should bear in mind two things. First, there is a gap here between precedents that are wrong and those that are *utterly* wrong. I want to leave open the possibility that, with respect to the latter, a commitment to the rule of law may not necessarily turn right what is utterly wrong—simply by following precedent.^72^ This caveat, however, does suggest that, with respect to less suboptimal precedents, or to precedents that do not necessarily reflect the later court’s preferred views, a commitment to the rule of law can do the justificatory work we are looking for.

Secondly, commitment to the rule of law does not necessarily ground a content-independent *obligation* to follow incorrectly decided precedents. It does, however, ground a content-independent *reason*—again, *pro tanto*. To say that courts *may* follow wrongly decided precedents in order to foster the rule of law is not to say that they *must*. It means only that we need to keep the door open for courts to reach legally justified decisions *even if* they follow precedents known to be wrong.

## 5. Two Modes of Precedential Reasoning

Commitment to the rule of law can make room for courts to adjudicate under robust precedent-following. Thus, the mere fact that an earlier court decided a dispute is a reason for the later court to follow this decision in a relevantly similar dispute. Under the strong version, the existence of a precedent-governed dispute triggers a non-contingent reason for action. The later court *always* has a reason to decide the same dispute by following precedent.

This non-contingent reason is a minimum requirement of the rule of law. Without it, we are susceptible of going back to the null model. But once this minimum requirement has been secured, there is yet another question that we need to ask ourselves. The answer to this question may divide legal systems. The question is whether the non-contingent reason to follow precedent should stay as a minimum requirement or should be reinforced. One way in which a legal system may reinforce its commitment to the rule of law is by requiring courts to treat the same reason as an *obligation*. For present purposes, an obligation is a requirement that derives from the combination of a first-order reason plus an exclusionary reason not to act on some conflicting reasons—a protected reason.^73^

Therefore, depending on a legal system’s commitment to the rule of law, we can identify two modes of precedential reasoning. One is characterised by the fact that the non-contingent reason to follow precedent is only a reason—and, as such, it can be outweighed by a more compelling reason. The other is characterised by that reason being a protected reason—ie having an exclusionary character—meaning later courts are prevented from acting on certain excluded reasons. Unlike the null model, these two modes are consistent with the rule of law—in fact, they are derived from this ideal.

In the following two subsections, I will articulate these modes in more detail. Following familiar terminology, I will call them ‘persuasive’ and ‘authoritative’.^74^ I will start with the latter, because it presents complexities worth disentangling from the outset. Before doing so, however, it is important to consider one important methodological caveat.

Whether an account is fully descriptive or may be detached, to a sensible extent,^75^ from a certain practice depends on the account’s purpose. In the introduction, I suggested that my aim is not to depict specific adjudicatory practices, for example, of either the civil law or the common law, but to provide a framework within which we may understand these practices. In particular, the authoritative mode aims to explain the idea of having a *protected reason* to follow precedent, while the persuasive mode involves that of having *a bare reason*. The authoritative mode, thus, is not necessarily a depiction of the common law, nor is the persuasive mode necessarily one of the civil law. Courts in these two traditions can have both types of reasons to follow precedent. Accordingly, the civil law may share features of the authoritative mode, while the common law may share ones of the persuasive mode.^76^

### A. The Authoritative Mode

Under the authoritative mode, whenever a precedent-governed dispute arises, the later court has a protected reason to follow precedent. This reason entails both a reason to decide the dispute in conformity with the precedent and an exclusionary reason *not to* decide the same dispute on certain reasons against following the precedent. If there are no non-excluded reasons against following precedent, then the later court *must* follow the relevant precedent—unless, as we will see, the precedent can be distinguished.^77^ Again, it is because of this combination of a first-order reason plus an exclusionary reason that we can think of the protected reason to follow precedent as giving rise to an *obligation*.^78^

Typically, the obligation of later courts to follow precedent applies whenever the precedent and present case are legally the same: whenever their similarities are relevant for the law, while their differences are not. When, conversely, these two cases are legally *different*, a later court may distinguish. In other words, the obligation of later courts under the authoritative mode has disjunctive form: either to follow or distinguish the precedent.^79^ ‘Distinguishing’, in particular, is the practice by which the later court shows that the present case has a novel fact not captured by the precedent, and which is legally relevant for handing down a different decision. In the common law, the majority of scholars think that distinguishing is an integral part of the practice of following precedent—and hence the obligation’s disjunctive form.^80^ Some scholars, however, think that later courts *should not* distinguish.^81^

This debate between common law scholars has one consequence for our analysis. What exactly does the obligation to follow precedent consist of? Is there an obligation for later courts to apply the legal rule provided by the *ratio decidendi* of the precedent? Or is such an obligation, as Grant Lamond claims, to treat the precedent as correctly decided on its facts?^82^ The fact that this debate is still ongoing makes it difficult to provide a clear-cut answer.^83^ My own view, which I hope to provide in detail soon, is that the obligation of a later court is to respect the decision of the earlier court to treat certain established facts as material, to give them a particular legal significance and to decide on the balance of reasons.^84^ This view would lean towards that of Lamond and later developed by John Horty.^85^ It can be summarised as follows: the later court must render a decision that is consistent with the authoritative treatment that the earlier court gave to the facts or factors of the precedent, as these were reported.

In any event, for now we need only to bear in mind that the specific content of the obligation to follow precedent is a matter of discussion in the common law. That obligation can take the form, *inter alia*, of having to apply the rule laid down by the *ratio*,^86^ or having to decide in a way that is consistent with the ‘background case’, as Horty puts it.^87^ But, to be sure, in both cases the later court has an obligation to decide the precedent-governed dispute by following precedent. In both cases, then, an authoritative mode exists. This mode, again, entails a reason for action—namely, decide the present case by following precedent—and an exclusionary reason *not to act* on certain conflicting reasons.^88^

One reason typically *excluded* from being acted upon by the later court is disagreeing with the precedent’s correctness. It is often the case that the later court must follow the relevant precedent even if it thinks that, by doing so, the court will render an otherwise suboptimal decision. In some exceptional cases, the legal system may authorise *some* courts to act on the otherwise excluded reason of disagreeing with the precedent’s substantive merits. When these precedents originated in the same court that may act on this *non*-excluded reason, the authorisation is typically known as the power to *overrule*. Its normative effect is twofold: for the later court, a reason otherwise excluded is a non-excluded reason. For the legal system, the overruled precedent loses its *erga omnes* force. The upshot is that such a precedent typically ceases to be part of the law.^89^

The grounds on which a court may overrule vary across jurisdictions—and also within one legal system. To illustrate: by virtue of the 1966 Practice Statement,^90^ the House of Lords (today the UK Supreme Court) is authorised to overrule its own precedents when ‘it appears right do so’. Accordingly, not following precedent when ‘it appears right to do so’ is a non-excluded reason.^91^ In contrast, in *Young v Bristol Aeroplane Co Ltd*,^92^ the Court of Appeal (Civil Division) declared itself to be bound by its own precedents, with three exceptions: (i) where two of these precedents would conflict with each other; (ii) where one of these precedents is incompatible with a decision of the House of Lords/Supreme Court; and (iii) where a precedent was given *per incuriam*. Thus, were the Court of Appeal to overrule one of its precedents because ‘it appears right to do so’ (as in the Practice Statement), that decision would be incompatible with the authoritative mode. The court would be acting on a reason that was excluded.^93^

A final remark. For various reasons, most of which have to do with effective action-guiding and better coordination between courts, a legal system may restrict the scope of the precedents that later courts must follow. Vertical and horizontal *stare decisis* are precisely ways to characterise this restriction. Now, the logic of grounding precedent-following in the rule of law does not necessarily preclude bottom-up *stare decisis*—ie higher courts being bound by the precedents of lower courts. However, by virtue of considerations such as effectiveness, this authoritative force is typically restricted to either vertical (top-down) or horizontal *stare decisis*. Yet one could still argue that the rule of law provides a non-contingent and *pro tanto* reason (albeit not a protected reason) for higher courts to consider at least some of the precedents of lower courts.

### B. The Persuasive Mode

Under the persuasive mode, whenever a precedent-governed dispute arises, the later court has a bare reason to decide the same dispute in conformity with the precedent. As such, this reason can be outweighed by a more compelling reason.

As suggested before, possibly a notable feature of the persuasive mode is that it is flexible, because it allows for different degrees of precedential constraint depending on the weight to be given to the reason in favour of following precedent. This reason could be very weighty if, say, the precedent originates from the highest tribunal in the land. Even in that scenario there might be room for various alternatives, depending on, say, whether the past decision was reached by a clear majority, a prestigious judge concurred and so on. By contrast, the same reason may be normatively weaker if, say, the precedent was reached by a lower court, or a court composed by one judge only.

Another factor giving more or less weight to the reason in favour of following precedent is whether the later court thinks that the precedent was rightly or wrongly decided. But this creates a problem: how can this substantive analysis be compatible with the content-*independent* character of the same reason? How can this reason allow for various weights depending on whether the later court agrees with the substance of the precedent?

This dilemma, to be sure, is merely apparent. Nothing in the notion of a content-independent reason prevents this reason from gaining *extra* weight by reference to some of its substantive merits.^94^ The idea of content independence guarantees the reason a normative minimum: a nucleus that cannot be reduced but which can become weightier. Thus, to the extent that the later court has a reason to follow precedent, that reason has a normative nucleus that will not be affected by whether the precedent was rightly or wrongly decided. On top of this nucleus, the later court may add extra weight if it considers that the dispute was, say, rightly decided, or it may add no further weight at all if the reverse holds.

As we can see, the structure of the persuasive mode allows room for various scenarios. One may argue that within this mode there exists a weak version in contrast to a strong one. Within the latter, certain courts will take very seriously the existence of a precedent regulating the case at hand, but will not see themselves as being bound by it. The former would make room for those judges for whom the reason to follow precedent can easily be outweighed by another, conflicting reason.

Finally, there are two features that distinguish the persuasive mode from the null model. First, in the persuasive mode, whenever a relevantly similar dispute arises, later courts *always* have a reason to follow precedent. This non-contingent reason does not exist in the null model. Secondly, the principles of judicial transparency and good government strongly militate in favour of courts adjudicating under the persuasive mode to explain why the reason to follow precedent has been outweighed. This is because, all else being equal, it is contrary to the rule of law to depart from relevant precedent without explaining why.

## 6. Evaluating the Modes

In this final section, I want to analyse how both modes of precedential reasoning deal with two evaluative questions. The purpose of this analysis is to offer *some* of the tools that are vital for determining whether there is a model that should be preferred, and eventually for criticising the particular practice of precedents held by courts.

### A. How Does Each Mode Advance the Rule of Law?

Let us start with stability and reliability. The authoritative mode often, but not necessarily, entails a view where precedents are part of the law. To the extent that courts are bound by precedent, those precedents may be treated as part of the law.^95^ Where this is so, every court with the power to set a precedent has also, in theory, the power to change the content of the law.^96^ Yet the authoritative mode reduces this margin for potential change by requiring later courts to decide the case at hand in conformity with the precedent. Thus, any time a precedent-governed dispute arises, the content of the law will remain somewhat stable, because the later court will have an obligation to follow precedent. This situation fosters reliability: people know in advance what courts have an obligation to do when they face a precedent-governed dispute.

On the persuasive mode, precedents are not, strictly speaking, part of the law, because they lack one essential feature that most legal norms (claim to) have: to provide authoritative guidance.^97^ Hence, it may seem that courts under this mode cannot change the content of the law when they hand down rulings. Although this is true, there can be grey areas, such as a legal system where the obligation of courts to follow precedent is not *de jure* but *de facto*.^98^ This situation can be problematic, for though, under a persuasive mode, the content of the law cannot be changed—again strictly speaking—by how courts *apply* the law, courts can nevertheless lead people to think that *applications* of the law are themselves part of the law. Courts, in other words, can affect reliability (what people may think the content of the law is) without changing the law itself.

This situation can be problematic because that *de facto* obligation depends mostly on contingencies of the legal system in question. It may depend, for example, on the particular composition of a court at a given time. Judges under this contingent composition may treat the reason in favour of following precedent as a protected reason. This, in turn, may create a contingent pattern of deciding precedent-governed disputes in conformity with the precedent. But because this pattern is the result of a *de facto* contingency and not of a *de jure* obligation, the pattern may change, such as when the composition of the court changes. In this new scenario, judges may treat the reason in favour of following precedent as a weak, non-protected reason. This can affect reliability, for people rely on this *de facto* pattern instead of relying on the law itself.^99^

Finally, in relation to equality, again the authoritative mode presents advantages *vis-à-vis* the persuasive mode. If the dispute at hand and the precedent are legally the same, then the later court has an obligation to hand down the same decision. In relation to the persuasive mode, there is no denying that if the dispute at hand and the precedent are legally the same, then both can be adjudicated alike. The problem, again, is that this adjudicatory treatment often follows as a matter of contingency, not obligation.

### B. How Does Each Mode Avoid Replicating Substantively Incorrect Decisions?

Suppose, to simplify the argument, that in 2019 the Supreme Court of an imaginary country handed down a decision that many respected judges and scholars consider mistaken on good grounds. How would both modes treat such a decision?

The authoritative mode, governing (typically, but not necessarily)^100^ the treatment that lower courts should give to the decision of the Supreme Court, would prevent these courts from rendering a decision different to that of the Supreme Court. For a lower court to hand down a substantively correct decision, the Supreme Court would have to overrule its precedent and issue a new decision that is substantively correct. This means that in those areas where the authoritative mode is in operation, lower courts can only reach substantively correct decisions contingently, as this correctness depends on whether the relevant precedents were correctly decided. If they were, then the later court would replicate a correct decision. If they were not, then the lower court would be required to replicate a substantively incorrect decision.^101^

Accordingly, there are three options a lower court has when faced with a wrongly decided precedent: to distinguish; to follow the precedent and render yet another substantively incorrect decision; or to adjudicate under the persuasive mode. If a later court wants to distinguish, as we saw above, then it must provide reasons showing that the case at hand presents a novel fact that is relevant for the decision. Sometimes, however, this fact does not exist. When this happens, the later court cannot distinguish, meaning it *must* replicate a decision that is substantively incorrect. This is the second option the court has, and seems to be the option the legal system wants the court to adopt when the precedent cannot be distinguished. Finally, the later court could adjudicate under the persuasive mode, but there might be responsibilities for doing so, particularly if that court has a legal duty to adjudicate under the authoritative mode.

It seems, therefore, that the authoritative mode places more value on advancing the rule of law than on deciding cases correctly, at least where this correctness entails contravening established precedent.^102^ By contrast, the weakness the persuasive mode showed in the last subsection can now be seen as virtues in relation to the value of deciding cases correctly. Under the persuasive mode, later courts do not have an obligation to follow precedent. Courts may outweigh the reason in favour of following precedent and act on a potentially more compelling reason, such as deciding the same dispute in a substantively correct way.^103^ This is not to deny the possibility that sometimes courts can have reasons as well for following wrongly decided precedents—this is why I argued in favour of robust precedent-following. For example, if the issue under consideration is so sensitive, reaching a different outcome may affect reliability in ways that are more prejudicial to the legal system than deciding the case in a substantively correct manner.

## 7. Conclusion

There is a minimum requirement of the rule of law in regard to precedent, and I have called it the persuasive mode of precedential reasoning. The null model, however lawful, runs counter to the rule of law. Yet the question still remains open as to which of the two modes that are compatible with the rule of law should be preferred. I do not think there is a unique answer here; it depends on the history of the legal system, its legal tradition and practices, and the values people in that system hold dear. Possibly in a legal system that attaches significant value to having democratic legislation as the main source of law, the reasons for leaning towards the persuasive mode are more demanding. For a system preferring flexibility in the adjustment of the law, the authoritative mode might be the best answer.

I have posed two evaluative questions, but much more could be said. For example, there are reasons to think that the degree of adjudicatory power that later courts have to give legal significance to the facts of the case varies between the two modes. In the authoritative mode, later courts cannot alter the legal significance that earlier courts gave to the facts of the precedent. In the persuasive mode, this obligation does not exist, at least not strictly speaking. Finally, there is the question of whether the authoritative mode entails judicial law making—at least in the robust form of a law-making power. There might be situations where indeed it does, but I will have to postpone that analysis for another time.

## Footnotes

1 Much of the literature has become increasingly technical, partly because of the valuable contributions of nonmonotonic logics and artificial intelligence. On the other hand, many scholars have focused exclusively on precedent in either the common law or the civil law. But even within one of these traditions we may find courts treating precedents differently. For example, an authoritative treatment in one common law country may appear as a persuasive treatment in another common law country.

2 I have borrowed part of this terminology from various contributions on the value of precedent-following. See eg D Lyons, ‘Formal Justice and Judicial Precedent’ (1985) 38 Vand L Rev 495; F Schauer, ‘Precedent’ (1987) 39 Stan L Rev 571, 595–602; L Alexander, ‘Constrained by Precedent’ (1989) 63 S Cal L Rev 1, 26 and 51; R Cross and JW Harris, *Precedents in English Law* (4th edn, Clarendon Press 1991) 11–12; N Duxbury, *The Nature and Authority of Precedent* (CUP 2008), ch 5; L Alexander and E Sherwin, *Demystifying Legal Reasoning* (CUP 2008) 35–7; J Waldron, ‘Stare Decisis and the Rule of Law: A Layered Approach’ (2012) 111 Mich L Rev 1, 31; N Varsava, ‘How to Realize the Value of Stare Decisis: Options for Following Precedent’ (2018) 30 Yale JL & Human 62, 70–3.

3 It goes without saying that this *ad hoc* terminology does not prevent *other* values from being part of the rule of law. The point is that, to the extent that there is considerable agreement in the literature in identifying stability, reliability and equality as rule-of-law values, we can call these values collectively—and for current purposes only—‘the rule of law’. cf text to nn 30, 31 and 32 below.

4 The names ‘authoritative’ and ‘persuasive’ are commonplace in the Anglo-Saxon literature on precedents, but their exact meanings vary. For ease of reference, I will follow this familiar terminology, hoping to provide a consistent understanding of what it means to treat precedents as either authoritative or persuasive. See n 74 below.

5 Many legal systems give *erga omnes* force to the decisions of specific courts. Thus, these rulings are universally authoritative—namely, they apply to all future cases that happen to be legally the same. Hence, we loosely say eg ‘this case is governed by precedent’. My notion of a precedent is prior to this universal authoritative force. A precedent may have *pro tanto* persuasive force, or it may have no force at all.

6 See K Stevens, ‘Reasoning by Precedent—Between Rules and Analogies’ (2018) 24 Legal Theory 216, 217.

7 The distinction between horizontal and vertical precedent does not play a crucial role in the valuable contributions of EM Wise, ‘The Doctrine of Stare Decisis’ (1975) 21 Wayne L Rev 1043; Lyons (n 2); Alexander (n 2); G Lamond, ‘Do Precedents Create Rules?’ (2005) 11 Legal Theory 1; J Horty, ‘Rules and Reasons in the Theory of Precedent’ (2011) 17 Legal Theory 1; Varasa (n 2); Stevens (n 6).

8 Horizontal *stare decisis* is the authoritative effect that precedents have on later courts of equivalent hierarchy. Vertical *stare decisis*, by contrast, is the authoritative effect that precedents of higher courts have on lower courts. In the literature, Frederick Schauer is one who has consistently maintained this distinction. Yet when it comes to the reasons for following precedent, he has generally focused on horizontal *stare decisis*—see eg Schauer (n 2) 578 fn 11; F Schauer, ‘Precedent’ in A Marmor (ed), *Routledge Companion to Philosophy of Law* (Routledge 2012) 130–2. In F Schauer, *Thinking Like a Lawyer* (Harvard UP 2012) 41–2, Schauer claims that ‘the justifications for precedential constraint [vertical *stare decisis*] are fairly obvious’: ‘lower court judges are expected to follow the “instructions” of those courts above them in what the military calls the “chain of command”’. This ‘chain of command’, however, is not obvious. The relation between higher and lower courts need not be of *adjudicatory* hierarchy—it could be of *administrative* hierarchy. But if a relation of adjudicatory hierarchy exists, it is not clear whether following precedent amounts to obeying a military command*.* Be that as it may, nothing in Schauer’s work suggests that the reasons for having horizontal *stare decisis* cannot support vertical *stare decisis* as well. For a somewhat sceptical analysis, in US law, of the arguments in favour of vertical *stare decisis*, see E Caminker, ‘Why Must Inferior Courts Obey Superior Court Precedents?’ (1994) 46 Stan L Rev 817.

9 J Gardner, *Law as a Leap of Faith* (OUP 2012) 192.

10 HLA Hart, *The Concept of Law* (3rd edn, Clarendon Press 2012) 7.

11 cf R Pound, ‘Justice According to Law’ (1913) 13 Colum L Rev 696.

12 In England, J Raz, *The Authority of Law* (2nd edn, OUP 2009) 184 fn 8 writes: ‘In English Law [the doctrine of precedent is] in fact part of the rule of recognition.’ This claim is extended to all common law countries by S Legarre and JC Rivera, ‘Naturaleza y dimensiones del “*stare decisis*”’ (2006) 33 Revista Chilena de Derecho 109, 112.

13 As one anonymous referee has correctly pointed out, many civil law countries have shown a tendency to depart from the classic civilian understanding of precedent to one where courts, at least in certain matters, are *required* to follow precedent. Good examples are Colombia, Mexico and Italy; see, respectively, C Bernal Pulido, ‘El precedente en Colombia’ (2008) 21 Revista Derecho del Estado 81; V Fernández Fernández, ‘La justicia de los precedentes. ¿Invasión a la independencia y autonomía del juzgador?’ (2016) XXIX Revista de Derecho (Valdivia) 9; A Cadoppi, *Il valore del precedente nel diritto penale* (2nd edn, Giappichelli Editore 2014). This tendency has led some scholars to consider where the civil law and the common law intersect; see Cadoppi (ibid.) 199. The tendency is undeniable, at least with respect to *some* civil law countries. But I would hesitate to call this tendency a full-blown system of *stare decisis*. To begin with, in many civil law countries the binding effect of precedents is usually reserved for the decisions of specific courts only—typically, higher courts and constitutional courts. Moreover, this effect is piecemeal, ie applicable to specific areas of law, such as constitutional law and criminal law. In many countries, furthermore, there is still considerable disagreement as to whether precedents *should* bind later courts—for a list of scholars who, in Spain, reject this view, see T García-Berrio Hernández, ‘La Controversia sobre el Precedente Judicial: un Clásico del Derecho en Constante Renovación’ (2006) 4 Foro, Nueva Epoca 127, 145–6. Given this disagreement and variety of practices, and also bearing in mind that the argument in this part is not about what the civil law is or is not today, I will rely on a rather classic approach to precedent-following in the civil law. The argument, to be sure, is about what we *may* learn from such an approach, namely that the null model is conceptually possible.

14 In Europe, examples of this relative force are provided by: art 1355 of France’s Code Civil; s 325(1) of Germany’s Zivilprozessordnung; and art 2909 of Italy’s Codice Civile. In Latin-America, similar examples can be found in: art 3 of Chile’s Código Civil; art 218 of Uruguay’s Código General del Proceso; and art 17 of Colombia’s Código Civil.

15 There are exceptions that could be classified as follows: (i) non-parties that may also benefit from the authoritativeness of the decision—eg lawful successors; and (ii) courts whose decisions bind *erga omnes*, eg constitutional courts.

16 E Steiner, *French Law: A Comparative Approach* (OUP 2010) 91 (emphasis in original). See also D Pugsley, ‘Two Systems of Precedent’ (1981) 15 The Law Teacher 7, 7: ‘Indeed French courts cannot even simply rely on a precedent; they must justify their own decisions themselves.’

17 JH Merryman and R Pérez-Perdomo, *The Civil Law Tradition* (4th edn, Stanford UP 2019) 84. In a similar vein, see Legarre and Rivera (n 12) 112; B Spaić, ‘The Authority of Precedents in Civil Law Systems’ (2018) XXVII Studia Iuridica Lublinensia 27, 28.

18 In Italy, see M Taruffo and M La Torre, ‘Precedents in Italy’ in N MacCormick and R Summers (ed), *Interpreting Precedents* (first published 1997, Routledge 2016) 155: ‘A judgment not applying a relevant precedent may be lawful in itself, since its lawfulness depends on its consistency with the statutory and constitutional provisions, not on its coherence with precedents.’

19 A Peczenik, ‘The Binding Force of Precedent’ in MacCormick and Summers (n 18) 463. Based on the example of Poland, Peczenik takes ‘further support’ to be cases where eg a precedent adds weight ‘to other legally relevant arguments for the decision handed down’; ibid 462.

20 If courts can lawfully adjudicate disputes without paying attention, in a normatively significant way, to relevant precedent, then it is doubtful whether something in law’s nature requires the practice of following precedent. It could be objected that nothing in the nature of the civil law would require this practice, but it would be part of the common law. Against this view, see Duxbury (n 2) 34, for whom ‘the common law does not need the doctrine of precedent in order to function’. cf Wise (n 7) 1057–8.

21 See eg Raz (n 12) 224; B Tamanaha, *On the Rule of Law. History, Politics, Theory* (CUP 2004) 33–4; J Waldron, ‘The Concept and the Rule of Law’ (2008) 43 Ga L Rev 1, 6; Waldron (n 2) 3–4.

22 This is the view associated with the well-known accounts of L Fuller, *The Morality of Law* (Yale UP 1969) ch 2; Raz (n 12) 213–14; J Finnis, *Natural Law and Natural Rights* (2nd edn, OUP 2011) 270–3; J Rawls, *A Theory of Justice* (first published 1971, Harvard UP 1999) 235–43. See also Tamanaha (n 21) 34, 66–7, 71, 93–4, 96 and 119; Waldron, ‘The Concept’ (n 21) 6; J Raz, ‘The Law’s Own Virtue’ (2019) 39 OJLS 1, 3–5.

23 See eg Tamanaha (n 21) 119; T Bingham, *The Rule of Law* (Allen Lane 2010) ch 9; Waldron, ‘The Concept’ (n 21) 7–8; J Waldron, ‘The Rule of Law and the Importance of Procedure’ (2011) 50 Nomos 3, 6.

24 See eg Tamanaha (n 21) 102–4 and 109–11; Bingham (n 23) ch 7; J Waldron, ‘The Rule of Law’, *The Stanford Encyclopedia of Philosophy* (Summer edn, 2020) <https://plato.stanford.edu/archives/sum2020/entries/rule-of-law/> accessed 20 June 2020.

25 See eg Tamanaha (n 21) 112–13.

26 See eg P Craig, ‘Formal and Substantive Conceptions of the Rule of Law: An Analytical Framework’ (1997) Public Law 467; Tamanaha (n 21) chs 7–9; Waldron, ‘The Rule of Law and the Importance of Procedure’ (n 23) 4–5; Waldron, ‘The Rule of Law’ (n 24); A Zanghellini, ‘The Foundations of the Rule of Law’ (2016) 28 Yale JL & Human 213, 214.

27 Tamanaha (n 21) 92.

28 ibid.

29 Gardner (n 9) 198–204.

30 A famous example of this disagreement can be found in Raz, *The Authority of Law* (n 12) 210–11. On the disputed nature of the rule of law, see J Waldron, ‘Is the Rule of Law an Essentially Contested Concept (in Florida)?’ (2002) 21 Law and Philosophy 137, 159; P Burgess, ‘The Rule of Law: Beyond Contestedness’ (2017) 8 Jurisprudence 480, 481.

31 Tamanaha (n 21) 102; similarly, ibid 111.

32 There is room in the literature for grounding precedent-following in more robust conceptions of the rule of law. The legal philosophy of Ronald Dworkin is one alternative. See R Dworkin, ‘Hard Cases’ (1975) 88 Harv L Rve 1057, 1087–101; R Dworkin, *Law’s Empire* (Harvard UP 1986) ch 7. A Dworkinian view of *stare decisis* was articulated by S Hershovitz, ‘Integrity and Stare Decisis’ in S Hershovitz (ed), *Exploring Law’s Empire: The Jurisprudence of Ronald Dworkin* (OUP 2006) ch 5. A critical examination of Dworkin’s views on precedent can be found in D Pannick, ‘A Note on Dworkin and Precedent’ (1980) 43 MLR 36. Discussing Dworkin’s views on precedent, see also S Perry, ‘Judicial Obligation, Precedent and the Common Law’ (1987) 7 OJLS 215, 223–6.

33 Other considerations may support following precedent—see eg Duxbury (n 2) ch 5; Varsava (n 2). Among them is the epistemic guidance that later courts may receive from knowing how earlier courts have dealt with statutes that are vague or have an unclear semantic meaning. Similarly, we may find arguments relying on the overall efficiency of the system’s adjudicatory process. I will not explore these other approaches, but I do not think my account is incompatible with them.

34 One may account for stability, reliability and equality in terms of rule-of-law values by looking at some of the most influential accounts of this ideal—see eg n 22 above.

35 Stability seems valuable only when we have *awareness* of something remaining stable and can thus be relied upon for future decision making. Now, because some may think that stability has value in its own right, I have decided to keep stability and reliability as separate values. Nothing in the main argument turns on this point. That stability is instrumentally valuable to reliability is a view present in T Benditt, ‘The Rule of Precedent’ in L Goldstein (ed), *Precedents in Law* (Clarendon Press 1987) 91; Duxbury (n 2) 160–1. Traces of this view can also be found from Lord Mansfield in Bingham (n 23) 38 and Hayek in Tamanaha (n 21) 66.

36 See eg C Perelman, *The Idea of Justice and the Problem of Argument* (J Petrie tr, Routledge & Kegan Paul 1963) 38; H Kelsen, *Pure Theory of Law* (M Knight tr, 2nd edn, University of California Pres 1967) 141–2; P Westen, ‘The Empty Idea of Equality’ (1982) 95 Harv L Rev 537, 551; J Raz, *The Morality of Freedom* (Clarendon Press 1986) 220–1.

37 On this point, see eg K Winston, ‘On Treating Like Cases Alike’ (1974) 62 CLR 1, 22; E Chemerinsky, ‘In Defense of Equality: A Reply to Professor Westen’ (1983) 81 Mich L Rev 575, 579–80.

38 On the various duties of judges, see eg L Green, ‘Law and the Role of a Judge’ in K Ferzan and S Morse (eds), *Legal, Moral, and Metaphysical Truths: The Philosophy of Michael S Moore* (OUP 2016) ch 22.

39 Winston (n 37) 18. Winston seems to have in mind *epieikeia*—equity, a corrective to law’s generality. See Aristotle, ‘Nicomachean Ethics’ in J Barnes (ed), *The Complete Works of Aristotle: The Revised Oxford Translation* vol II (Princeton UP 1984) 1796 (1137^b^17–18).

40 The requirement of justification is particularly stringent when the relevant party has showed to the court that there is precedent regulating the matter at hand, but also if it happens that the later court is aware of this precedent—or others that are relevant.

41 Raz, ‘The Law’s Own Virtue’ (n 22) 15.

42 Finnis (n 22) 270.

43 Fuller (n 22) 33.

44 Raz, *The Authority of Law* (n 12) 229; Gardner (n 9) 192; L Green, ‘Should Law Improve Morality?’ (2013) 7 Criminal Law and Philosophy 473, 487.

45 Bingham (n 23) 8.

46 That is, I first articulated the null model by looking at specific features of the civil law tradition and then criticised it on the basis of the rule of law.

47 On the idea of a second-order reason, see text to nn 64, 65 and 66 below.

48 Some of which is provided in n 2 above; but see in particular Duxbury (n 2) 159: ‘The point … is not that precedent-following is supportable because stability and closure are intrinsically good objectives, but that if judges consider these objectives desirable then precedent-following is one way by which they can pursue them.’

49 See Lyons (n 2) 511–12; Schauer, ‘Precedent’ (n 2) 597–8; Duxbury (n 2) 163.

50 Or part of an exceptional set of decisions. This suggests that fostering the rule of law is more a matter of degree than of kind, insofar as a legal system may tolerate exceptions to the various rule of law *desiderata*. cf T Endicott, *Vagueness in Law* (OUP 2000) 191.

51 As Schauer, ‘Precedent’ (n 2) 598 puts it: ‘maintaining a serious regime of precedential constraint entails some number of suboptimal decisions’. In a similar vein, see R Kozel, *Settled Versus Right: A Theory of Precedent* (CUP 2017) 6.

52 A Marmor, ‘Should Like Cases Be Treated Alike?’ (2005) 11 Legal Theory 27, 29.

53 [1966] 1 WLR 1234 (emphasis added).

54 cf Hart (n 10) 128; L Duarte d’Almeida, *Allowing for Exceptions* (OUP 2015) 184.

55 According to Duxbury (n 2) 128, ‘only rarely since 1966 has the House of Lords used the power that it created for itself’. On the House of Lord’s Practice Statement, see ibid 123–49; W Twining and D Miers, *How to Do Things with Rules* (5th edn, CUP 2010) 283–5.

56 Such as a significant number of courts in the common law tradition.

57 cf n 51 above; Stevens (n 6) 219.

58 K Stevens, ‘Case-to-Case Arguments’ (2018) 32 Argumentation 431.

59 Raz, *The Morality of Freedom* (n 36) 388; R Chang, ‘Hard Choices’ (2017) 3 Journal of the American Philosophical Association 1, 17.

60 This distinction is based on Stevens, ‘Case-to-Case Arguments’ (n 58) 435–7, who distinguishes between weak and strong versions of the principle ‘treating like cases alike’. Now, one should be careful not to conflate precedent-following with treating like cases alike or to ground the former in the latter. Both have been a debated topic in the literature. See eg Lyons (n 2); F Schauer, ‘On Treating Unlike Cases Alike’ (2018) 33 Constitutional Commentary 437.

61 Schauer, ‘Precedent’ (n 2) 575; cf Schauer, *Thinking Like a Lawyer* (n 8) 90; Varsava (n 2) 76; Stevens (n 6) 219; Kozel (n 51) 6.

62 Stevens, ‘Case-to-Case Arguments’ (n 58) 436–7.

63 ibid 437.

64 See J Raz, *Practical Reasons and Norms* (first published 1975, OUP 1999) 39.

65 ibid; Raz, *The Authority of Law* (n 12) 17.

66 Raz, *The Authority of Law* (n 12) 18.

67 Chang (n 59) 17 (emphasis added).

68 Raz, *The Morality of Freedom* (n 36) 388.

69 This proviso is important. It means that there are no relevant differences between the two cases that would warrant a different treatment.

70 Duxbury (n 2) 128.

71 This commitment may well constitute part of the duty that judges assume when they are sworn in. I thank Crescente Molina for this suggestion.

72 cf L Green, ‘Law as a Means’ in P Cane (ed), *The Hart–Fuller Debate in the Twenty-First Century* (Hart Publishing, 2010) 22; Green, ‘Law and the Role of a Judge’ (n 38) 335.

73 See text to n 66 above.

74 See n 4 above. In my view, what is either authoritative or persuasive is not the precedent itself—a precedent is a non-normative fact. Only by virtue of a moral consideration can a precedent be a *pro tanto* normative fact. That is why I prefer to speak of the *modes of precedential reasoning* as being either authoritative or persuasive.

75 As a minimum methodological requirement, any sound account needs to pick central features of the practice it wishes to analyse; see Lamond (n 7) 15; A Rigoni, ‘Common-Law Judicial Reasoning and Analogy’ (2014) 20 Legal Theory 133, 135–6; Stevens, ‘Reasoning by Precedent’ (n 6) 218–19. But once these central features are picked, there is room for a sensible detachment from practice—see n 76 below.

76 The methodological approach I have adopted is vital for sorting out possible factual counterexamples. To be sure, any descriptive account of what courts do—eg how they reason using precedents—is potentially subject to counterexamples. Consider the following case: Perry (n 32) 230–4 attributed to Raz a ‘positivistic’ account of precedent-following—one where later courts have exclusionary reasons to follow precedent. He then criticised this positivistic framework on grounds of descriptive inaccuracy—many common law courts distinguish in ways that are incompatible with Raz’s proposal. For example, they drop conditions for the *ratio*’s application—ibid 239. In a nutshell, according to Perry, legal positivism (at least as presented by Raz) fails to account for what many common law courts do when they reason using precedents. Perry then offered another possible understanding of the practice*.* But Perry’s objection would misfire if there are some common law courts in the world that treat precedents as giving rise to exclusionary reasons. His objection would backfire if his own account is affected by a set of plausible counterexamples. Both options are always possible, given the large field of ongoing adjudication. A sensible detachment from practice is beneficial because it allows us to identify *modes of precedential reasoning* that can exist independently of a particular legal tradition.

77 Stevens, ‘Reasoning by Precedent’ (n 6) 218 characterises the situation of a later court that has not found a non-excluded reason against following precedent as having a *binding* reason to follow precedent. When, by contrast, such a court has an *authoritative* reason, the court can either follow or distinguish the precedent by acting on a non-excluded reason.

78 For current purposes, we can follow Raz on this point; see eg J Raz, ‘Promises and Obligations’ in PMS Hacker and J Raz (ed), *Law, Morality, and Society: Essays in Honour of HLA Hart* (Clarendon Press 1977) 223, arguing that ‘An action is obligatory if it is required by a categorical rule, ie a mandatory rule which applies to its subjects not merely because adherence to it facilitates achievement of their goals’. Later, in Raz, *The Authority of Law* (n 12) 234–5, Raz seemed to refer to such a categorical rule in terms of a protected reason: ‘I have argued elsewhere [ie the work just cited] that “an action is obligatory only if it is required by a protected reason”.’

79 Raz, *The Authority of Law* (n 12) 185; Lamond (n 7) 3; Schauer, *Thinking Like a Lawyer* (n 8) 58.

80 See eg Lamond (n 7) 15, arguing that ‘Any satisfactory view of precedent must see distinguishing as an integral part of it rather than as an add-on or quirk of the common law’.

81 Notably Alexander and Sherwin (n 2) 122: ‘The rule model of judicial decision making has no conceptual room for the practice of distinguishing rules’; ibid 123: ‘distinguishing rules is logically equivalent to repealing rules at will’.

82 Lamond (n 7) 1, 3, 16, 23 and 25.

83 See Stevens, ‘Reasoning by Precedent’ (n 6) for the current state of this debate.

84 By treating certain established facts as material, I mean making them relevant for deciding the present case. By giving a particular legal significance, I mean deciding whether a specific fact, previously treated as material by the court, favours one of the parties. By deciding on the balance of reasons, I mean establishing the necessary and sufficient conditions for reaching a justified decision.

85 Horty (n 7).

86 There is significant literature on the various tests for discovering the *ratio decidendi* of a case. Much of this literature dates back to the first half of the twentieth century—and even before. By then, as is well known, legal realists like H Oliphant and JF Francis offered revolutionary views about the *ratio* of a precedent—many of them attacking its supposed determinacy. Authors such as Arthur Goodhart offered tests where the purpose was to make the *ratio* more determinate, so that the distinction between the *ratio* and the *obiter* of a case may be useful in explaining precedential constraint. I unfortunately cannot address this vast literature here. For a helpful study, see Cross and Harris (n 2) ch II; Duxbury (n 2) 67–92; Twining and Miers (n 55) 304–12.

87 Horty (n 7) 13.

88 In relation to the common law, Lamond (n 7) 19 attempts more precision by saying that ‘the *ratio* would provide the basis for the first-order part of the protected reason. The other facts of the case would provide the basis for the exclusionary reason; later courts are excluded from relying on reasons provided by features that were present in the precedent case to defeat the first-order reason for the result.’ Recently, Robert Mullins, ‘Protected Reasons and Precedential Constraint’ (2020) 26 Legal Theory 40 has defended an account of what he calls ‘the protected reason model of precedent’. See eg ibid 52–3: ‘On the protected reason model developed in the previous section, a past decision binds a subsequent decision-maker when the rule relied on in the previous decision is applicable to the factual scenario that applies in the subsequent case. The decision-maker must take the ratio of the previous decision as a reason for deciding in the same way and a reason for excluding otherwise applicable reasons present in these past cases from acting as the basis for an inconsistent judgment.’

89 With the proviso of n 95 below. There is also the question of whether a precedent could lose its *erga omnes* force prospectively *only*. See Twining and Miers (n 55) 285 fn 48, discussing *Re Spectrum Plus Ltd* [2005] UKHL 41, where the House of Lords concluded that, in order to do so, it would have to be an exceptional case.

90 See text to n 53 above.

91 See Twining and Miers (n 55) 284 for some instances in which the House of Lords has acted on this non-excluded reason.

92 [1944] KB 718.

93 The argument needs to be qualified. According to Twining and Miers (n 55) 286–7, the Court of Appeals has restated aspects of the doctrine laid down in *Young v Bristol Aeroplane*, allowing for departures from relevant precedent when ‘the previous decision is manifestly wrong’.

94 I think G Lamond, ‘Persuasive Authority in the Law’ (2010) XVII Harvard Review of Philosophy 16, 17 would agree with me: ‘Obviously the second approach [ie that certain sources have content-independent normative weight] does not rule out considering the persuasiveness of the view. *The view could carry even more weight where it is found to be convincing*’ (emphasis added).

95 Strictly speaking, however, authority and legal validity are very different ideas. Whether a norm is *authoritative* depends—at least for current purposes—on whether it provides a protected reason for action; see eg Raz, *The Authority of Law* (n 12) 21–5. By contrast, whether a norm is *legal* depends on whether it meets the criteria for legal validity given by the rule of recognition. This makes room for the possibility that a precedent could be ‘authoritative’ (in a loose sense—see n 74 above) without being part of the law. But we need not make too much out of this conceptual possibility. Raz, *The Authority of Law* (n 12) has argued that, at least in England, the rule of recognition gives legal validity to authoritative precedents. There is no reason to think that the same could happen with respect to *other* legal systems—cf Legarre and Rivera (n 12).

96 cf Raz, *The Authority of Law* (n 12) 185; Lamond, ‘Do Precedents Create Rules?’ (n 7) 17; Gardner (n 9) 80.

97 See eg Raz, *The Authority of Law* (n 12) ch 2. An excellent elaboration of law’s claim to authority can be found in Gardner (n 9) ch 5. The qualification ‘strictly speaking’ (in the text) is important because there could be a grey area, which I cannot explore here, where norms that provide a reason only, as opposed to a protected reason, could nonetheless become *legal* norms. This will depend on other, possibly complementary jurisprudential views about law’s authoritativeness. See eg A Marmor, ‘Soft Law, Authoritative Advice and Non-binding Agreements’ (2019) 39 OJLS 507.

98 In relation to the civil law, see Merryman and Pérez-Perdomo (n 17) 47; Peczenik (n 19) 461 and 465–6. I believe a real tension may exist between *de jure* and *de facto* precedent-following when a statutory provision explicitly provides that judicial decisions are binding on the parties to the dispute *only*—as in Chile; see n 14 above.

99 There is no denying that the law may offer less guidance, and thus affect reliability, without authoritative applications of it binding later courts.

100 See the final remark in section 5A.

101 As correctly noted by Duxbury (n 2) 24, Waldron, ‘Stare Decisis’ (n 2) 8 and Stevens, ‘Reasoning by Precedent’ (n 6) 219, all writing in relation to the common law.

102 cf G Postema, ‘Some Roots of our Notion of Precedent’ in Goldstein (n 35) 31.

103 In relation to the civil law, see Merryman and Pérez-Perdomo (n 17) 85.

